# Aflatoxin B1 Exacerbates Genomic Instability and Apoptosis in the BTBR Autism Mouse Model via Dysregulating DNA Repair Pathway

**DOI:** 10.3390/toxics11070636

**Published:** 2023-07-22

**Authors:** Ali A. Alshamrani, Mohammad Y. Alwetaid, Mohammed A. Al-Hamamah, Mohamed S. M. Attia, Sheikh F. Ahmad, Majed A. Algonaiah, Ahmed Nadeem, Mushtaq A. Ansari, Saleh A. Bakheet, Sabry M. Attia

**Affiliations:** Department of Pharmacology and Toxicology, College of Pharmacy, King Saud University, Riyadh 11451, Saudi Arabia; aaalshamrani@ksu.edu.sa (A.A.A.); mywetaid@gmail.com (M.Y.A.); mohammad_9119@hotmail.com (M.A.A.-H.); mohamed.sabre2023@gmail.com (M.S.M.A.); fashaikh@ksu.edu.sa (S.F.A.); malgonaiah@ksu.edu.sa (M.A.A.); anadeem@ksu.edu.sa (A.N.); muansari@ksu.edu.sa (M.A.A.); sbakheet@ksu.edu.sa (S.A.B.)

**Keywords:** autism, food pollutants, DNA damage, DNA repair, carcinogenesis

## Abstract

The pathophysiology of autism is influenced by a combination of environmental and genetic factors. Furthermore, individuals with autism appear to be at a higher risk of developing cancer. However, this is not fully understood. Aflatoxin B1 (AFB1) is a potent food pollutant carcinogen. The effects of AFB1 on genomic instability in autism have not yet been investigated. Hence, we have aimed to investigate whether repeated exposure to AFB1 causes alterations in genomic stability, a hallmark of cancer and apoptosis in the BTBR autism mouse model. The data revealed increased micronuclei generation, oxidative DNA strand breaks, and apoptosis in BTBR animals exposed to AFB1 when compared to unexposed animals. Lipid peroxidation in BTBR mice increased with a reduction in glutathione following AFB1 exposure, demonstrating an exacerbated redox imbalance. Furthermore, the expressions of some of DNA damage/repair- and apoptosis-related genes were also significantly dysregulated. Increases in the redox disturbance and dysregulation in the DNA damage/repair pathway are thus important determinants of susceptibility to AFB1-exacerbated genomic instability and apoptosis in BTBR mice. This investigation shows that AFB1-related genomic instability can accelerate the risk of cancer development. Moreover, approaches that ameliorate the redox balance and DNA damage/repair dysregulation may mitigate AFB1-caused genomic instability.

## 1. Introduction

Autism is a neurodevelopmental disorder, classically observed in the first three years of lifetime, that can cause impairments in social interactions and communication [1]. The prevalence of autism has increased dramatically in recent decades, and it is estimated that 1 in every 100 children globally has autism, with a sex ratio of 4:1 for boys to girls [2]. Individuals with autism often have several comorbidities, such as genetic metabolic diseases, genetic syndromes, and cancer [3]. The cause of autism remains unclear; nevertheless, both environmental and genetic components are assumed to play substantial roles. Numerous genetic alterations have been related to patients with and without autism, and many of the altered genes in autistic patients are related to DNA repair [4,5,6]. Disturbances in the DNA repair pathway can affect repair accuracy, leading to the generation of unrepaired DNA damages and, consequently, cancer. Indeed, elevated oxidative DNA damages have been observed in individuals with autism and autism animal models [7,8]. Furthermore, the lack of effective repair in autism can accelerate DNA damage [6,9].

Alterations in the DNA damage/repair pathway can induce variations in repair proficiency, leading to greater sensitivity to carcinogenic pollutants. Autism is thus linked to an elevated tumor risks; for example, breast, brain, and ovarian tumors are more prevalent in patients with autism than in those without [10,11]. Elevated cancer risk in patients with autism is suggested to be linked to the inadequacy of DNA repair-related genes. Genomic sequencing has shown a wide interference in the risk genes for autism and malignancy [4], indicating that the increased tumor risk in autistic individuals may have a robust genetic basis. Several of these altered genes have been associated with the pathogeneses of malignancy development, involving genes included in DNA damage and repair [10,11].

Autism symptoms can be identified in early childhood; however, autism is frequently not diagnosed until much later. As diagnostic methods have advanced, the prevalence of autism has risen considerably in recent decades [12]. The available scientific research suggests that there are likely many factors that trigger the disease and make a child more likely to have autism, including environmental factors. Recent reports agree that genomic susceptibility to different contaminants, such as foods, may be involved in the elevated risk of neurodevelopmental diseases due to the presence of xenobiotics [13,14]. Xenobiotics in foods are recognized as risk factors for autism, and it has also been postulated that they may contribute to disease severity. Mycotoxin is a xenobiotic whose induced toxicity may be remarkably related to the characterization of autistic disorders, as it can modify neurological and immune systems, produce oxidative stress, and aggravate damage to the intestinal barriers [15,16].

Mycotoxins are pollutants formed by ubiquitous fungi in a wide range of foods under precise humidity and temperature conditions, both on farms and during storage. Their harmful effects are a concern for animal and human health, as exposure can occur with food intake but also from inhalation in environmentally polluted regions [17]. Associations between numerous mycotoxins and various health complications, including developmental and neurological impacts, have been published in both animal and human experiments [18,19,20]. Among the reported mycotoxins, aflatoxin B1 (AFB1) reportedly has the most harmful effects and is the most potent identified nutritional carcinogen, as it induces cancers in numerous organs in the human body [21]. AFB1 is primarily metabolized into reactive mutagenic intermediates that induce the formation of AFB1/DNA adducts and oxidative DNA strand breaks, consequently increasing the risk of cancer [22]. As several different harmful effects caused by mycotoxin exposure are similar to those often linked to autism as comorbidities [15,16,23], it was hypothesized that autism comorbidities could be exacerbated by the harmful effects of AFB1 pollutants.

AFB1 has been shown to disrupt the blood–brain barrier, and chronic AFB1 exposure can lead to AFB1 accumulation in human and animal brain tissues, suggesting that AFB1 may directly harm various brain cells [24,25]. Autism is thought to be a developmental disorder caused by hippocampal dysfunction [1]. Memory, spatial reasoning, and social interaction are all supported by the hippocampus and are impaired in autism [26]. Recent data showed that chronic exposure to AFB1 increases hippocampal microglial pyroptosis and vulnerability to stress in mice [25], suggesting that the hippocampus is most affected by chronic AFB1 exposure. Although mycotoxins are among the pollutants suggested to be risk factors for autism, existing information is limited and debatable. Italian studies have reported that mycotoxins reverse some genes related to autism, with sex-specific toxicological effects in men [27,28]. Although these investigations observed a substantial relationship between mycotoxins in subjects with autism compared to healthy subjects, other investigations did not detect this relationship [29].

Preliminary data from our laboratory show that AFB1 increases the severity of neurobehavioral and immune disturbances in BTBR mice (a model for autism), which is associated with the aggravation of autism-like behavioral phenotypes (Alwetaid MY., submitted). The effects of AFB1 on genomic instability and apoptosis in autism, however, have yet to be studied. As a result, we wanted to determine if AFB1 exposure induces changes in genomic stability and apoptosis in BTBR mice. Such studies are important because the genomic instability induced by harmful pollutants can cause mutations, and if these cells continue to proliferate, the risk of cancer increases.

## 2. Materials and Methods

### 2.1. Mice

Male BTBR and C57BL/6 (B6) animals that were 8–9 weeks old and 20–26 g each were used in this investigation. All animals were ordered from the Jackson Laboratory (Bar Harbor, ME, USA) and housed in clean boxes in a room that was 21 ± 2 °C, with approximately 50% relative humidity and a 12 h light-dark cycle, and standard animal food and water were accessible ad libitum. The experiments were carried out according to the Institutional Animal Care and Use Committee of KSU, SA (approval number KSU-SE-22-81).

### 2.2. Treatment

Aflatoxin B1 (AFB1; Sigma-Aldrich, St. Louis, MO, USA) was dissolved in corn oil and given daily via oral gavage at 1.25 mg/kg for 28 consecutive days, while control mice received only corn oil. The received volume was 100 μL per 10 g. Ethylnitrosourea (ENU) at 50 mg/kg was intraperitoneally injected once as a positive mutagen [30]. The repeated tested dose of AFB1 is non-lethal in mice (LD_50_ = 48.79 mg/kg) [31] and no dead mice were observed in this study after repeated exposure to the AFB1. Animals were sacrificed 24 h following the last oral dose of AFB1, then brain tissues and bone marrow cells were sampled. Marrow cells from both femurs were transferred into tubes containing 1000 μL of calf serum.

### 2.3. Unrepaired DNA Damage Estimates

Unrepaired DNA damage was estimated using a bone marrow micronucleus assay, as previously described [32]. Isolated cells were smeared on glass slides and stained with Giemsa–May–Grunwald solutions. For each animal, 4000 polychromatic erythrocytes (PCEs) on a coded slide were examined for the micronuclei (MN) [33]. Moreover, the number of PCEs amongst 1.000 normochromatic erythrocytes (NCE) per mouse was calculated to evaluate bone marrow suppressions as follows: % PCE = [PCE/(PCE + NCE)] × 100.

### 2.4. DNA Strand Break Estimations

Spontaneous DNA strand break was estimated using an alkaline comet test based on OECD guidelines, as detailed previously [34]. Bone marrow cells were diluted with low-melting-point agarose and smeared on pre-coated slides with normal-melting-point agarose. After gel solidification on ice, coded slides were dipped in the lysing solution at 4 °C. After 12 h, slides were submerged in cold electrophoresis buffer for 20 min for DNA unwinding. Electrophoresis, neutralization, and ethidium bromide staining were then performed as described previously [35]. Two coded slides were made for each mouse, and a minimum of 150 nuclei were analyzed on each slide with the Comet Assay IV (Perceptive Instruments, UK). The percentage data for tail intensity were used to estimate the level of spontaneous DNA damage.

### 2.5. DNA Repair Enzyme Incubation

A modified comet test using DNA repair endonucleases was also performed on slides in parallel to the alkaline comet test to determine the presence of oxidized purine and pyrimidine nucleotides [36]. Following the lysis step, slides were rinsed with enzyme buffer and then digested with 50 µL of Fpg and Endo III enzymes (which recognize strand DNA breaks at altered purine and pyrimidine, respectively) or incubated in endonuclease buffer at 37 °C for 30–45 min [37]. After endonuclease digestion, the slides were submerged in an electrophoresis buffer and electrophoresed, neutralized, stained, and analyzed as described above for the alkaline comet test. The occurrence of oxidative DNA strand break was determined as the difference in tail intensity between endonuclease and buffer incubations.

### 2.6. Oxidant and Antioxidant Balance

Brain samples were collected to estimate lipid peroxidation and GSH levels. The concentration of malondialdehyde (MDA) formed via oxidative damage of lipids was determined by making thiobarbituric acid-reactive substance estimations, according to Ohkawa’s procedure [38], using freshly prepared 1,1,3,3-tetra-methoxy-propane as a standard [39]. GSH level was estimated with 5,5′ -dithiobis-(2-nitrobenzoic acid) based on the procedure of Ellman [40], using GSH standard solutions [41]. The results are shown as μmol/g protein. Protein was determined using a BCA protein assay kit (Santa Cruz Biotechnology, Dallas, TX, USA) with calf serum albumin (BSA) as the standard and according to the supplier’s procedure.

### 2.7. Apoptosis Estimation

Phosphatidylserine on the surface of the induced apoptotic bone marrow cells was estimated using Annexin V/propidium-iodide (PI) staining and the activity of caspase-3, which distinguishes the DEVD sequence and was estimated using the Caspase-3 assay kit, as previously described [42]. Apoptosis was assessed by determining the movement of phosphatidylserine to the outer cell surface using an Annexin V/FITC Kit (BioVision, Mountain View, CA, USA) according to the supplier’s procedure. The stained cells were measured using a FACSCalibur flow cytometer (Becton Dickinson, San Jose, CA, USA) at 488/530 nm for Annexin V/FITC (FL1) and 488/585 nm for PI staining (FL2). The activity of caspase-3 was estimated with ELISA using the Caspase-3 colorimetric assay kit (BioVision, Mountain View, CA, USA), according to the manufacturer’s protocol [43]. The formation of the chromophore pNA after cleavage from the labeled DEVD-pNA substrate was detected at 405 nm using a microtiter plate reader.

### 2.8. RT-PCR Analysis

The expression of the Bax, Gadd45a, p53, Xrcc1, and Ogg1 genes were quantified in the brain tissue samples using RT-PCR, respectively. Total RNA was isolated using an RNeasy Mini Kit (Qiagen, Valencia, CA, USA), as described by the manufacturer and used for cDNA syntheses [44]. The expression of the tested mRNA and the housekeeping gene Cyclophilin A in the cDNA were all estimated using primers synthesized by GenScript (Table 1). Quantitative RT-PCR was carried out using SYBR^®^ Green on an ABI 7500 RT-PCR System (Applied Biosystems, Austin, TX, USA). The relative expression was calculated using the 2^−ΔΔC^T method [45].

## 3. Results

### 3.1. AFB1 Exacerbates Unrepaired DNA Break in BTBR Animals

**Figure 1:**
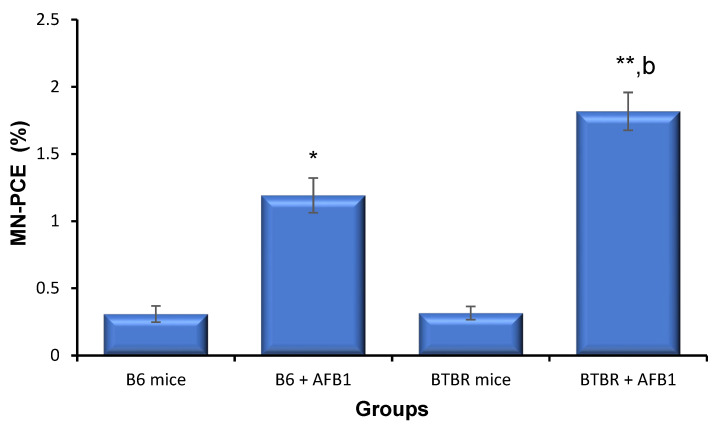
Frequencies of micronucleated polychromatic erythrocytes (MN-PCE) in the bone marrow of mice 24 h after their last exposure to aflatoxin B1 (AFB1, 1.25 mg/kg for 28 days; mean ± SD). * *p* < 0.05, ** *p* < 0.01 vs. B6 control mice (Kruskal–Wallis test). ^b^ *p* < 0.01 vs. B6 + AFB1 (Mann–Whitney *U*-test).

The MN percentage was statistically higher in the positive control than the vehicle control B6 mice (1.51 ± 0.26). The BTBR mice did not demonstrate any statistically differences in the incidence of MN when compared to the vehicle control B6 (Figure 1), while for the mice treated with AFB1, there was a statistically significant stimulatory effect on MN generation with both the BTBR and vehicle control B6 when compared to the unexposed control B6. Furthermore, the frequency of MN in the BTBR animals exposed to AFB1 was statistically higher than that in the AFB1-exposed B6 animals (*p* < 0.01). In the present investigation, no bone marrow suppression was observed and the PCE ratios were similar in all mice.

### 3.2. AFB1 Exacerbates Oxidative DNA Damage in BTBR Mice

**Figure 2:**
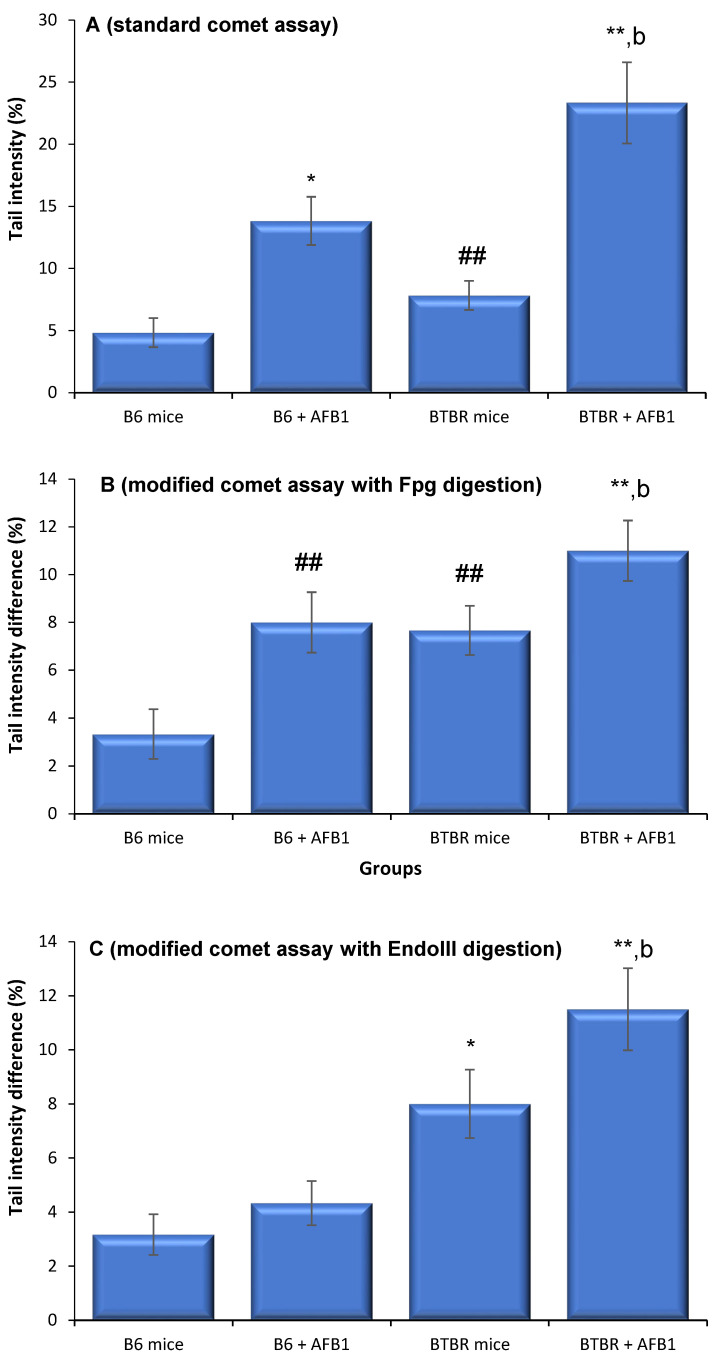
Levels of DNA strand breaks (tail intensity) in the bone marrow of mice 24 h after their last exposure to aflatoxin B1 (AFB1, 1.25 mg/kg for 28 days; mean ± SD). (**A**) = cells incubated with buffer only, (**B**) = cells incubated with Fpg, and (**C**) = cells incubated with Endo III. * *p* < 0.05, ** *p* < 0.01 vs. B6 control mice (Kruskal–Wallis test). ^##^ *p* < 0.01 vs. B6 mice and ^b^ *p* < 0.01 vs. B6 + AFB1 (Mann–Whitney *U*-test).

The level of DNA damage in the B6 mice administrated ENU was statistically higher than that of the vehicle control B6 animals (21.6 ± 3.8; *p* < 0.01). The level of spontaneous DNA strand break in the BTBR was significantly different when compared with the vehicle control B6 animals, demonstrating that there was DNA damage (Figure 2A). A statistical change was also observed in both BTBR and B6 animals after AFB1 administration when compared to the vehicle control B6; AFB1-exposed BTBR animals also exhibited a greater level of spontaneous DNA damage than the AFB1-exposed B6 animals (*p* < 0.01).

The incubation of bone marrow cells with DNA repair enzymes induced a statistically increase in the percentage of spontaneous DNA damage in all prepared slides when compared to the slides incubated with enzyme buffer alone, demonstrating the presence of oxidized pyrimidine and purine bases in the bone marrow cells sampled from the B6 and BTBR mice. Following digestion with the Endo III and Fpg enzymes, a statistical increase in DNA damage was seen in cells sampled from the ENU-injected B6 animals [(Endo III (19.3 ± 3.0 and Fpg (18.3 ± 2.3); *p* < 0.01]. Incubation of the slides with Fpg statistically elevated the proportion of oxidative DNA damage in the B6 mice samples after exposure to AFB1 (Figure 2B). In contrast, incubation with Endo III resulted in a weak and non-significant increase in the oxidative DNA strand breaks (Figure 2C). These data suggest that AFB1 mainly induces DNA lesions sensitive to Fpg digestion in B6 animals. In contrast, the BTBR mice samples exposed to AFB1 had more DNA damage after the slides were incubated with both endonucleases than both the BTBR and B6 unexposed and B6 AFB1-exposed mice.

### 3.3. AFB1 Exacerbates the Oxidant and Antioxidant Imbalance in BTBR Animals

The concentration of MDA in the BTBR mice was statistically higher than that in the vehicle control B6 animals (*p* < 0.05; Table 2). A statistical rise in MDA concentration was also seen in AFB1-treated BTBR and B6 mice compared with the MDA concentration in the vehicle control B6 animals (*p* < 0.01). Furthermore, the BTBR mice exposed to AFB1 showed a higher concentration of MDA than the B6 mice exposed to AFB1 (*p* < 0.01). There was a statistical decrease in GSH concentration in the AFB1-treated and untreated BTBR animals when compared with the control B6 group, and AFB1-exposed BTBR animals presented a greater decrease in GSH than the AFB1-exposed B6 animals (*p* < 0.01).

### 3.4. AFB1 Exacerbates Apoptosis in BTBR Mice

**Figure 3:**
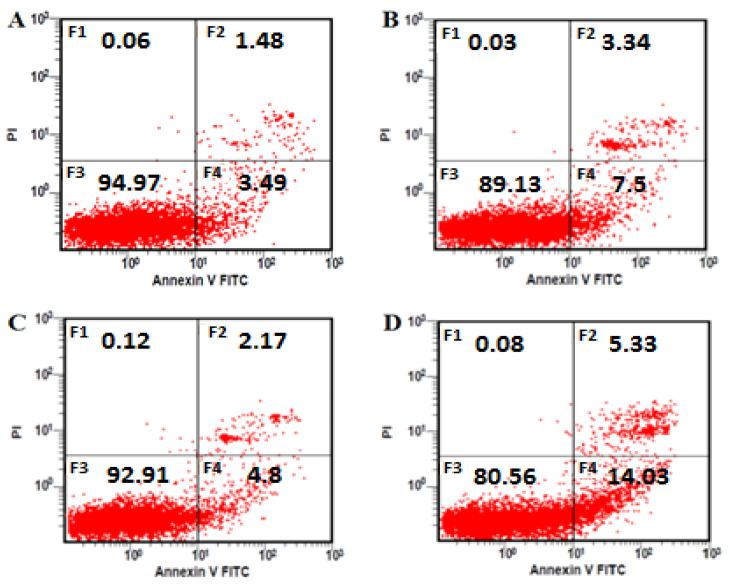
A representative flow cytometry image of Annexin V-propidium iodide (PI) double-stained bone marrow cells of mice 24 h after their last exposure to aflatoxin B1 (AFB1, 1.25 mg/kg for 28 days; mean ± SD). (**A**) = B6 control mice, (**B**) = B6 mice treated with AFB1, (**C**) = BTBR mice, and (**D**) = BTBR mice treated with AFB1.

**Figure 4:**
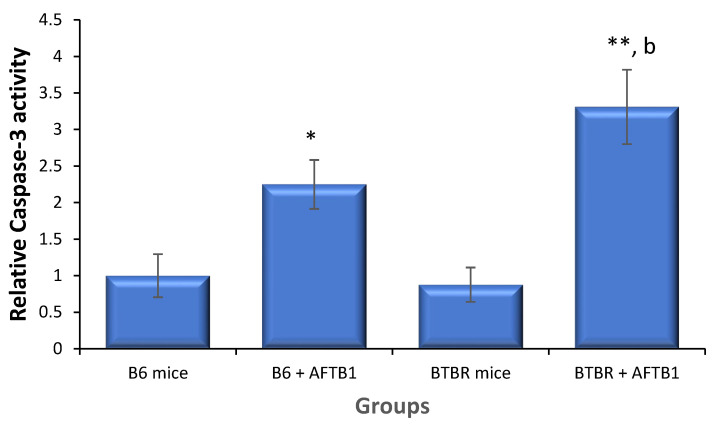
Relative caspase-3 activity in the bone marrow cells of mice 24 h after their last exposure to aflatoxin B1 (AFB1, 1.25 mg/kg for 28 days; mean ± SD). * *p* < 0.05, ** *p* < 0.01 vs. B6 control mice and ^b^ *p* < 0.01 vs. B6 + AFB1 (ANOVA test).

Representative images of Annexin V-PI flowcytometric analysis are presented in Figure 3. The incidence of apoptotic bone marrow cells did not differ between the BTBR and vehicle control B6 animals (Table 3). On the other hand, the incidence of apoptotic cells in the BTBR and B6 animals exposed to AFB1 was statistically greater than that in the vehicle control B6 animals (*p* < 0.01), and the BTBR animals treated with AFB1 showed a greater percentage of early apoptotic cells than AFB1-exposed B6 animals (*p* < 0.01). To study the participation of the caspase 3 lysosomal protein in signal transductions, the hydrolytic activity of caspase 3 towards DEVD pNA was quantified using ELISA. There were no apparent differences observed in the protein activity between BTBR mice and vehicle-treated control B6 animals (Figure 4). However, the caspase 3 activity was statistically elevated in BTBR and B6 mice following exposure to AFB1. The BTBR animals exposed to AFB1 showed caspase-3 activity that was significantly increased relative to that in the B6 mice exposed to AFB1 (*p* < 0.01).

### 3.5. AFB1 Exacerbates Gene Expression Alterations in BTBR Animals

**Figure 5:**
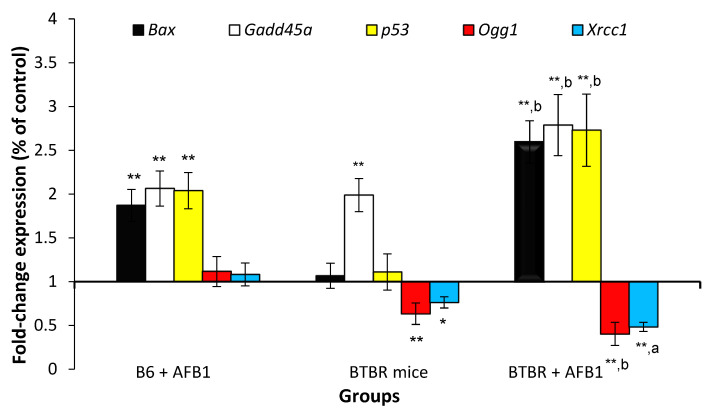
RT-PCR analysis of the brain tissues from mice 24 h after their last exposure to aflatoxin B1 (AFB1, 1.25 mg/kg for 28 days; mean ± SD). * *p* < 0.05, ** *p* < 0.01 vs. B6 control mice and ^a^ *p* < 0.05, ^b^ *p* < 0.01 vs. B6 + AFB1 (ANOVA test).

The expression changes in the *Bax, Gadd45a*, *p53*, *Ogg1*, and *Xrcc1* mRNA transcripts relative to the vehicle control B6 mice are shown in Figure 5. Significant upregulation in the expression of the *Bax, Gadd45a*, and *p53* mRNA transcripts was observed after AFB1 exposure in B6 mice when compared to the vehicle control B6 animals. No apparent alteration was detected in the expression of the *p53* and *Bax* mRNA transcripts between the BTBR and vehicle control B6 mice. However, an obvious upregulation in the expression of the *Gadd45a* mRNA transcript and downregulation of the *Ogg1* and *Xrcc1* mRNA transcripts was observed, indicating DNA repair impairment in the BTBR animals. The expression levels of the *Bax*, *Gadd45a*, and *p53* mRNA transcripts were statistically augmented in AFB1-treated BTBR animals compared to the unexposed BTBR mice and B6 mice exposed to AFB1. Furthermore, the administration of AFB1 to BTBR animals reduced the downregulation of the *Xrcc1* and *Ogg1* mRNA transcripts to levels that were significantly different from those in the unexposed BTBR animals and B6 mice exposed to AFB1.

## 4. Discussion

The generation of DNA strand breaks and decrease in repair efficiency following exposure to the environmental pollutant AFB1 have been proposed as the main determinants of sensitivity to AFB1-evoked carcinogenesis [46,47]. The influences of repeated exposure to AFB1 on genomic instability and apoptosis in autism have not yet been studied. Therefore, we aimed to examine whether repeated exposure to AFB1 causes alterations in the oxidant/antioxidant balance and DNA repair system and, consequently, genomic instability, a hallmark of cancer and apoptosis in the BTBR autism mouse model. The results of our investigation have revealed that mice exposed to AFB1 had greater genomic instability and apoptosis than control B6 animals. Furthermore, the BTBR mice exposed to AFB1 showed significantly higher levels of genomic instability and apoptosis than B6 mice exposed to AFB1. These data suggest that aggravated genomic instability and apoptotic effects in BTBR animals may be due to AFB1 exposure. Elevated MN generation and oxidative DNA damage were detected in BTBR mice exposed to AFB1. AFB1-treated B6 animals also showed increased MN generation and oxidative DNA damage; nevertheless, the magnitude was much lower than that in AFB1-treated BTBR animals. The data verify that BTBR animals are susceptible to pollutants [9,48].

Increased MN incidence is a predictive biomarker of cancer risk [49]. In the current investigation, no apparent change was seen in the incidence of MN between BTBR and vehicle control B6 animals. This is in agreement with earlier investigations of blood samples collected from patients with autism. By matching lymphoblasts from individuals with autism and their relatives, Fenech et al. found no variation in micronuclei generation, demonstrating that subjects with autism do not suffer from gross alterations in their DNA structures [50]. Furthermore, in our earlier investigation of isolated lymphocytes from children with autism, we did not detect any changes in the occurrence of metaphase chromosome aberrations [51]. When BTBR animals were treated with AFB1, a significant increase in MN formation was observed when compared to the vehicle control B6 animals. Moreover, a significant rise in the frequency of MN formation was detected in the B6 animals exposed to AFB1, which verifies the reported positive association between the MN generation and exposure to AFB1 in animals and humans [52,53]. Nevertheless, BTBR mice were more sensitive to AFB1 and AFB1-exposed BTBR animals, presenting a statistically greater level of MN generation than AFB1-exposed B6 animals.

Previous reports have stated an overall increase in spontaneous DNA damage in both autistic patients and BTBR animals [7,9,51]. In harmony with these studies, the present results show elevated DNA damage in BTBR animals and increased susceptibility to damage in animals exposed to AFB1. DNA damage in response to AFB1 exposure has previously been described and is thought to arise through either reduced repair efficiency and/or direct oxidation of DNA strands because of disturbed DNA repair pathways [54]. The detected hypersusceptibility of animals with autism to AFB1-induced DNA damage is consistent with the proposed role of certain genetic differences in repairing damaged DNA in patients with autism [4,10]. Following the incubation of bone marrow cells with the DNA repair endonucleases Fpg and Endo III, a higher level of oxidative DNA damage was observed, and BTBR mice exposed to AFB1 presented greater DNA damage than both unexposed BTBR and B6 animals exposed to AFB1. This shows that oxidative modifications in DNA nucleotides are important for the DNA damage that is detected in BTBR animals after exposure to AFB1.

Elevated levels of oxidative stress were reported in autistic animals [9,55,56]. This effect can cause DNA damage due to the impairment of the redox system, which occurs because the endogenous antioxidant GSH can detoxify and protect cells from the deleterious effects of the reactive species produced by xenobiotics. The results indicate that AFB1-treated BTBR animals have increased oxidative stress, as demonstrated by their lipid peroxidation, with a decrease in the GSH levels greater than those in AFB1-exposed B6 animals, which may be due to the ability of AFB1 to enhance reactive oxygen species generation. AFB1 exposure produces reactive species in exposed cells both in vitro and in vivo [57]. Thus, AFB1 could increase total oxidative strand breaks in BTBR animals via an oxidant/antioxidant imbalance and subsequently accelerate genomic instability and apoptosis in autism.

Previous studies have shown that AFB1 triggers apoptosis in both animal and human cells [57,58]. The activation of programmed cell death in mice, as demonstrated by Xu et al., revealed that elevated levels of Bax, p53, and caspases stimulated apoptosis in the liver of AFB1-exposed mice [57]. In addition, Liu and colleagues reported that AFB1 stimulates the expression of Bax and p53, and thus the activation of caspase-3 in human hepatocyte [58]. The data of our investigation correlate with earlier studies, as the expression of Bax, p53, and caspase 3 activity increased, resulting in the activation of the apoptotic pathway. In our investigation, we did not observe any variations in the basal levels of apoptotic cells in the BTBR animals. On the other hand, AFB1 markedly elevated the percentage of apoptotic bone marrow cells in the BTBR and B6 animals, as identified through Annexin V and PI staining; this effect was exacerbated in BTBR mice treated with AFB1. Similarly, caspase-3 activity was statistically elevated in both B6 and BTBR animals following AFB1 exposure. Furthermore, caspase-3 activation was augmented in BTBR animals exposed to AFB1 compared with that of B6 mice exposed to AFB1.

DNA repair enzymes eliminate the most damaging errors generated by ordinary metabolic actions and environmental factors. When the level of damaging errors is high and repair is inadequate, apoptosis-triggering downstream signaling effectors are stimulated, causing cell death [59]. An AFB1-produced mutation in DNA repair genes, such as p53, may elevate genomic damage in cells chronically exposed to AFB1 [60]. Numerous animal investigations have linked impairments in the repair pathway to autism [4,8,10]. In alignment with these investigations, our expression results revealed a statistical downregulation of the expression of the *Ogg1* and *Xrcc1* repair genes in BTBR animals compared with that in vehicle control B6 animals. Altered expression of DNA repair genes was observed in all animals exposed to AFB1. However, BTBR animals were more susceptible to the changes caused by AFB1 in the *Gadd45a*, *Bax*, *p53*, *Ogg1*, and *Xrcc1* genes than the B6 animals exposed to AFB1. This suggests that insufficient repair of the damaged DNA may be a vital mechanism in the exacerbation of genomic instability and apoptosis in BTBR animals following AFB1 exposure. AFB1 could thus exacerbate oxidative DNA strand breaks in BTBR mice through redox disturbances and alterations in DNA repair, consequently exacerbating genomic instability and apoptosis.

## 5. Conclusions

The results of this investigation demonstrate for the first time that exposure to the pollutant AFB1 significantly exacerbates oxidative stress and changes the expression of DNA damage/repair genes, which increases the sensitivity of autistic animals to AFB1 toxicity, reduces DNA repair efficiency, and aggravates genomic instability and apoptosis. The observed alterations in genomic stability, a hallmark of cancer and apoptosis in autistic animals, warrant future large-scale studies to specifically address the role of alterations in DNA damage/repair gene expression in the pathogenesis of autistic disorders in the human population. These findings indicate the need for safety and prophylactic management to help prevent late health toxicity due to AFB1 exposure. Patients with autism should thus receive continuous medical follow-ups to facilitate the early detection of genomic alterations. Approaches that ameliorate redox imbalances and DNA repair alterations may also mitigate AFB1-induced genomic instability and apoptosis.

## Figures and Tables

**Table 1:** Primers used for RT-PCR.

Gene	Forward Primer	Reverse Primer
*Bax*	5’-ATGGAGCTGCAGAGGATCAT-3’	5’-GATCAGCTCGGGCACTTTAG-3’
*Gadd45a*	5′-TGCGAGAACGACATCAACAT-3′	5′-TCCCGGCAAAAACAAATAAG-3′
*p53*	5’-CACAGCGTGGTGGTACCTTA-3’	5’-TCTTCTGTACGGCGGTCTCT-3’
*Ogg1*	5’-GATTGGACAGTGCCGTAA-3’	5’-GGAAGTGGGAGTCTACAG-3’
*Xrcc1*	5’-CAGACAGCACACATCTCATC-3’	5’-ACCCTCCTCAGTTCATCCT-3’
*Cyclophilin A*	5’-TGGTCAACCCCACCGTGTTCTTCG-3’	5’-TCCAGCATTTGCCATGGACAAGA-3’

**Table 2:** Concentrations of lipid peroxidation (MDA) and reduced glutathione (GSH) in the brain tissues of mice 24 h after their last exposure to aflatoxin B1 (AFB1, 1.25 mg/kg for 28 days; mean ± SD).

Groups	MDA (μmol/g Protein)	GSH (μmol/g Protein)
B6 mice	0.72 ± 0.19	13.16 ± 2.78
B6 + AFB1	1.40 ± 0.25 **	7.33 ± 1.63 **
BTBR mice	1.24 ± 0.20 *	9.16 ± 1.17 *
BTBR + AFB1	2.11 ± 0.36 **^,b^	4.83 ± 1.60 **^,b^

* *p* < 0.05, ** *p* < 0.01 vs. B6 control mice and ^b^ *p* < 0.01 vs. B6 + AFB1 (ANOVA test).

**Table 3:** Frequency of apoptotic bone marrow cells in mice 24 h after their last exposure to aflatoxin B1 (AFB1, 1.25 mg/kg for 28 days; mean ± SD).

Groups	Live Cells (%)	Early Apoptotic Cells (%)	Late Apoptotic and Necrotic Cells (%)	Damaged Cells (%)
B6 mice	94.82 ± 2.63	2.99 ± 0.59	1.6 ± 0.69	0.57 ± 0.35
B6 + AFB1	88.18 ± 4.22 *	7.25 ± 0.75 **	4.22 ± 1.10 *	0.33 ± 0.16
BTBR mice	93.99 ± 1.99	3.18 ± 1.12	2.16 ± 0.56	0.65 ± 0.34
BTBR + AFB1	79.77 ± 5.84 **^,b^	15.19 ± 1.93 **^,b^	4.47 ± 1.45 *	0.56 ± 0.33

* *p* < 0.05, ** *p* < 0.01 vs. B6 control mice and ^b^ *p* < 0.01 vs. B6 + AFB1 (ANOVA test).

## Data Availability

The authors confirm that all data underlying the findings are fully available without restriction. All relevant data are within the paper.
